# Kuwanon T and Sanggenon A Isolated from *Morus alba* Exert Anti-Inflammatory Effects by Regulating NF-κB and HO-1/Nrf2 Signaling Pathways in BV2 and RAW264.7 Cells

**DOI:** 10.3390/molecules26247642

**Published:** 2021-12-16

**Authors:** Wonmin Ko, Zhiming Liu, Kwan-Woo Kim, Linsha Dong, Hwan Lee, Na Young Kim, Dong-Sung Lee, Eun-Rhan Woo

**Affiliations:** 1 College of Pharmacy, Chosun University, Dong-gu, Gwangju 61452, Korea; rabis815@naver.com (W.K.); lzmqust@126.com (Z.L.); donglinsha011@163.com (L.D.); ghksdldi123@hanmail.net (H.L.); 2 Department of Herbal Crop Research, National Institute of Horticultural and Herbal Science, RDA, Eumseong 27709, Korea; swamp1@naver.com; 3 Pathology Research Division, National Institute of Fisheries Science, Busan 46083, Korea; pharm001@korea.kr

**Keywords:** *Morus alba*, kuwanon T, sanggenon A, BV2, RAW264.7 cells, anti-inflammatory effects

## Abstract

We previously investigated the methanolic extract of *Morus alba* bark and characterized 11 compounds from the extract: kuwanon G (**1**), kuwanon E (**2**), kuwanon T (**3**), sanggenon A (**4**), sanggenon M (**5**), sanggenol A (**6**), mulberofuran B (**7**), mulberofuran G (**8**), moracin M (**9**), moracin O (**10**), and norartocarpanone (**11**). Herein, we investigated the anti-inflammatory effects of these compounds on microglial cells (BV2) and macrophages (RAW264.7). Among them, **3** and **4** markedly inhibited the lipopolysaccharide (LPS)-induced production of nitric oxide in these cells, suggesting the anti-inflammatory properties of these two compounds. These compounds inhibited the production of prostaglandin E2, interleukin-6, and tumor necrosis factor-α, and the expression of inducible nitric oxide synthase and cyclooxygenase-2 following LPS stimulation. Pretreatment with **3** and **4** inhibited the activation of the nuclear factor kappa B signaling pathway in both cell types. The compounds also induced the expression of heme oxygenase (HO)-1 through the activation of nuclear factor erythroid 2-related factor 2. Suppressing the activity of HO-1 reversed the anti-inflammatory effects caused by pretreatment with **3** and **4**, suggesting that the anti-inflammatory effects were regulated by HO-1. Taken together, **3** and **4** are potential candidates for developing therapeutic and preventive agents for inflammatory diseases.

## 1. Introduction

*Morus alba*, a medicinal plant belonging to the family Moraceae, has been used for treating pulmonary inflammatory conditions in traditional medicine [1]. Its root bark contains various active components such as umbelliferone, scopoletin, mucins, tannins, flavonoids (morusin, mulberrin, mulberrichromene, cyclomulberrin, moracin P, moracin O, mulberrofuran Q, kuwanon E, and kuwanon H) [2], 2-arylbenzofurans (moracenin D, moracin P, moracin O, and mulberrofuran Q), and prenylated flavonoids (licoflavone C, cyclomulberrin, neocyclomorusin, sanggenon I, morusin, and kuwanon U) [3,4]. *M. alba* extracts and active compounds can alleviate lung diseases, as revealed by recent studies [5,6,7]. In addition to the pharmacological effect of *M. alba* on the lungs, isoprenylated flavonoids (sanggenol Q, kuwanon T, sanggenon N, mulberrofuran G, and mulberrofuran C) exert hepatoprotective effects in t-BHP-induced HepG2 cells [8]. Prenyl-flavonoids (kuwanon A, kuwanon C, kuwanon T, and morusin) and triterpenoids (betulic acid, uvaol, and β-sitosterol) substantially inhibit the differentiation of 3T3-L1 adipocytes [9]. Morin hydrate, the major flavonoid constituent of *M. alba*, alleviates chronic unpredictable stress-induced memory impairment, indicating that this compound could boost the antioxidant defense system and inhibit neuroinflammatory pathways [10]. These studies suggest that the constituents contained in *M. alba* are potential candidates for treating various diseases.

Inflammation, a complex self-defense response to injurious stimuli, plays an important role in immune defense through the activation of several immune cells, including macrophages, monocytes, leukocytes, mast cells, and other cell types [11]. Macrophages are the most abundant and widely distributed immune cells in the body, and microglia are resident macrophages in the central nervous system (CNS) [12]. Macrophages and microglial cells are key players of the inflammatory response. They can be activated in response to stimuli such as lipopolysaccharide (LPS), cytokines, and chemokines, and induce inflammatory conditions [13]. Under inflammatory conditions, excessively activated macrophages and microglial cells cause abnormal regulation of pro-inflammatory mediators (nitric oxide (NO), prostaglandin E2 (PGE_2_), inducible nitric oxide synthase (iNOS), and cyclooxygenase (COX)-2) and pro-inflammatory cytokines (interleukin (IL)-6 and tumor necrosis factor (TNF)-α), through the activation of the nuclear factor kappa B (NF-κB) signaling pathway [14,15]. Increased levels of pro-inflammatory mediators further exacerbate the progression of inflammatory diseases, which further increase activation of inflammatory factors, thereby resulting in a vicious cycle [16]. Therefore, regulating inflammatory mediators is essential for the treatment and prevention of inflammatory diseases.

Heme oxygenase (HO)-1 is a rate-limiting enzyme catalyzing the degradation of heme into biliverdin, ferrous ion (Fe^2+^), and carbon monoxide (CO) [17]. HO-1 induction is regulated by the activation of nuclear factor erythroid 2-related factor 2 (Nrf2). HO-1 can be induced in response to oxidative stress and inflammation to protect tissues and maintain homeostasis in the body [18]. Therefore, targeting HO-1 induction is one of the potential strategies for treating inflammatory diseases.

In our ongoing strive for exploring candidate(s) from natural products to treat inflammatory diseases, we herein present the anti-inflammatory effects of 11 compounds isolated from *M. alba* in LPS-induced BV2 and RAW264.7 cells.

## 2. Results

### 2.1. Effects of 11 Compounds Isolated from M. alba on the Viability of BV2 and RAW264.7 Cells

**Figure 1:**
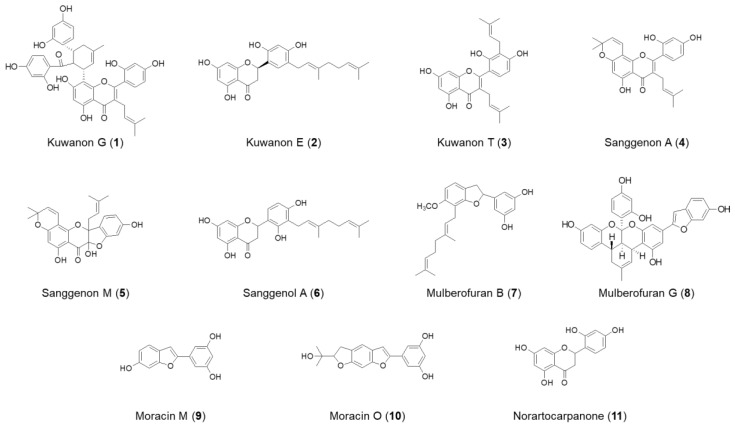
Chemical structures of compounds **1**–**11** isolated from *M. alba*.

**Figure 2:**
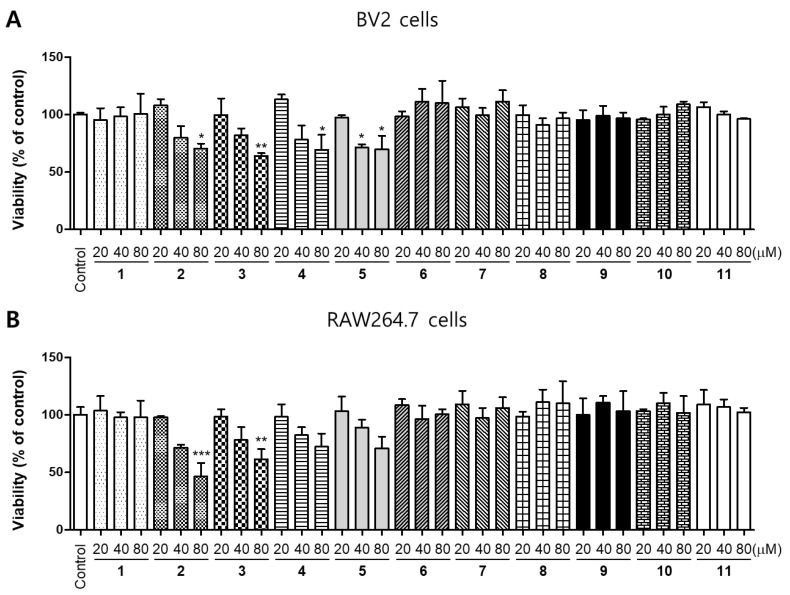
Cytotoxic effects of compounds **1**–**11** isolated from *M. alba* on BV2 (**A**) and RAW264.7 (**B**) cells. The cells were incubated for 48 h with various concentrations of the compounds, and their viability was determined using MTT (3-(4,5-dimethylthiazol-2-yl)-2,5-diphenyltetrazolium bromide) assay. Error bars represent mean ± standard deviation of three independent experiments. * *p* < 0.05, ** *p* < 0.01, *** *p* < 0.001 compared with the control group.

In the previous study, the root bark of *M. alba* was extracted in aqueous methanol, and the obtained extracts were successively partitioned into EtOAc, n-BuOH, and H_2_O. Repeated SiO_2_, ODS, and Sephadex LH-20 column chromatography of the EtOAc fraction afforded 11 compounds, such as kuwanon G (**1**), kuwanon E (**2**), kuwanon T (**3**), sanggenon A (**4**), sanggenon M (**5**), sanggenol A (**6**), mulberofuran B (**7**), mulberofuran G (**8**), moracin M (**9**), moracin O (**10**), and norartocarpanone (**11**) [6,19,20]. The chemical structures of 11 compounds isolated from *M. alba* are illustrated in Figure 1. To determine the cytotoxic effects of these compounds, a 3-(4,5-dimethylthiazol-2-yl)-2,5-diphenyltetrazolium bromide (MTT) assay was performed, BV2 and RAW264.7 cells were treated with the indicated concentrations of the compounds for 48 h, at concentrations of up to 80 μM. (Figure 2). Compounds **2**, **3** and **4** had toxic effects at 80 μM, compound **5** had toxic effects at 40 μM. Based on the result of the toxicity evaluation, a non-toxic concentration range was selected for subsequent studies on the anti-inflammatory effects (compound **2**, **3**, **4** at 40 μM, compound **5** at 200 μM, and other compounds at 80 μM).

### 2.2. Effects of 11 Compounds Isolated from M. alba on the Expression of Inflammatory Factors and iNOS Protein in BV2 and RAW264.7 Cells

**Figure 3:**
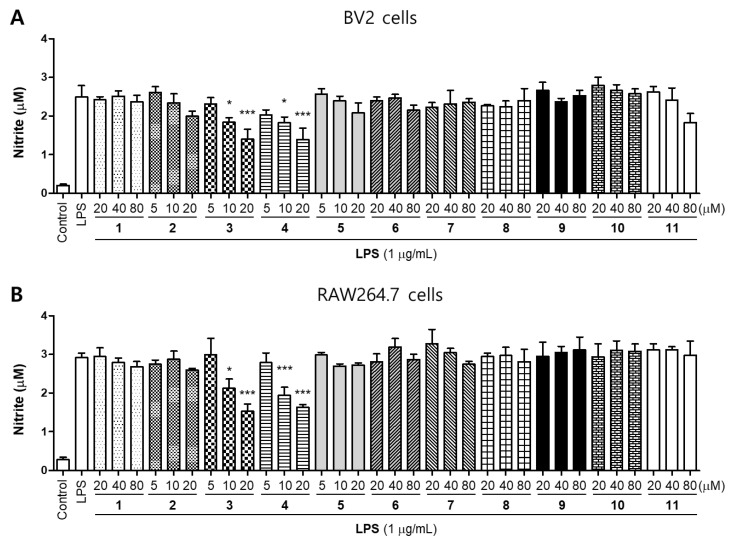
Inhibitory effects of compounds **1**–**11** on nitrite production in BV2 (**A**) and RAW264.7 (**B**) cells. The cells were pretreated for 2 h with concentrations of compounds and stimulated for 24 h with lipopolysaccharide (LPS; 1 μg/mL). Error bars represent mean ± standard deviation of three independent experiments. * *p* < 0.05 and *** *p* < 0.001 compared with the LPS-treated group.

NO is a free radical in the cardiovascular, nervous, and immune systems. It maintains intracellular homeostasis, transports neurotransmitters, and regulates anti-inflammatory activity and cytotoxicity. However, when a large amount of NO is produced, it has detrimental effects on the body, including vasodilation, cytotoxicity, and tissue damage [21,22]. We then examined the effects of these compounds on nitrite production in LPS-induced BV2 and RAW264.7 cells. Cells were treated with different concentrations of compounds for 2 h prior to stimulation with LPS (1 μg/mL) for 24 h. Among the compounds, only Compound **3** (kuwanon T) and **4** (sanggenon A) significantly inhibited nitrite production in both BV2 and RAW264.7 cell lines (Figure 3). In addition, we conducted an additional experiment to compare the inhibitory effect of nitrite production between the LPS-treated group after the compound pretreatment and the compound-treated group after the LPS pretreatment. As a result, there was no difference on the inhibitory effect of nitrite production between the two experimental groups (Supplementary Figure S1). Therefore, in this study, the following experiments were conducted using the pre-treatment with compounds within 2~3h before treating LPS.

**Figure 4:**
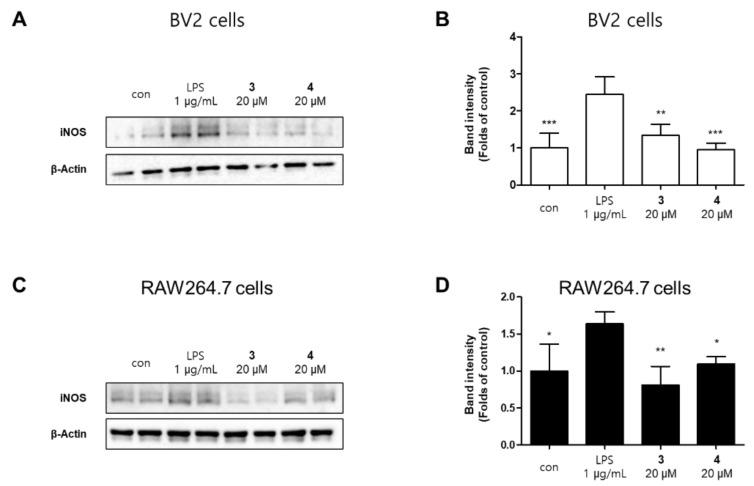
Protein expression levels of inducible nitric oxide synthase (iNOS) in lipopolysaccharide (LPS)-stimulated BV2 (**A**) and RAW264.7 (**C**) cells. The cells were pretreated for 2 h with various concentrations of compounds **3** or **4** and stimulated for 24 h with LPS (1 μg/mL). Representative blots from three independent experiments are shown. Immunoblots were quantified using the ImageJ software. Band intensities are normalized to that of β-actin (**B**,**D**). Error bars represent mean ± standard deviation of three independent experiments. * *p* < 0.05, ** *p* < 0.01, and *** *p* < 0.001 compared with the LPS-treated group.

**Figure 5:**
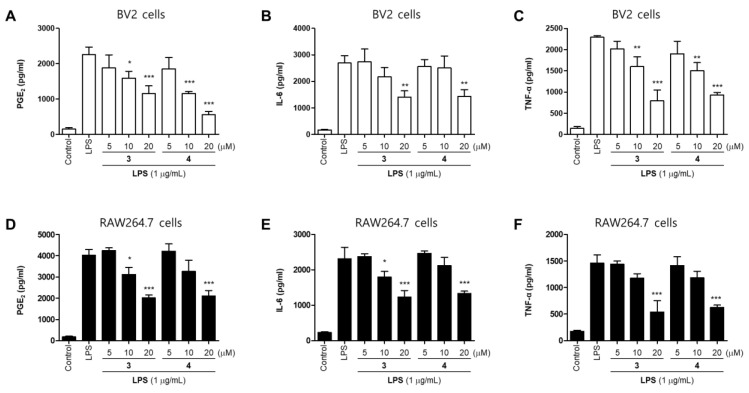
Inhibitory effects of compounds **3** and **4** on the level of PGE_2_ (**A**,**D**), IL-6 (**B**,**E**), and TNF-α (**C**,**F**) in BV2 and RAW264.7 cells. The cells were pretreated for 2 h with various concentrations of compounds **3** or **4** and stimulated for 24 h with lipopolysaccharide (LPS; 1 μg/mL). Error bars represent mean ± standard deviation of three independent experiments. * *p* < 0.05, ** *p* < 0.01 and *** *p* < 0.001 compared with the LPS-treated group.

The production of NO increased by the pro-inflammatory proteins inducible nitric oxide synthase (iNOS). However, overexpression of iNOS seriously impairs the pathophysiology of the disease [23]. Compound **3** and **4** inhibited the expression of iNOS in a concentration-dependent manner (Figure 4).We examined the effects of compounds **3** and **4** on LPS-induced expression of inflammatory factors in BV2 and RAW264.7 cells. The cells were treated with different concentrations of compounds **3** and **4** for 2 h prior to stimulation with LPS (1 μg/mL) for 24 h. Both compounds significantly inhibited LPS-induced expression of PGE_2_, TNFα, and IL-6 in BV2 and RAW264.7 cells (Figure 5). The results appeared to show that pre-treatment with compound **3** and **4** suppressed the LPS-induced inflammation in BV2 and RAW264.7 cells.

### 2.3. Effects of Compounds 3 and 4 on NF-κB Translocation in BV2 and RAW264.7 Cells

**Figure 6:**
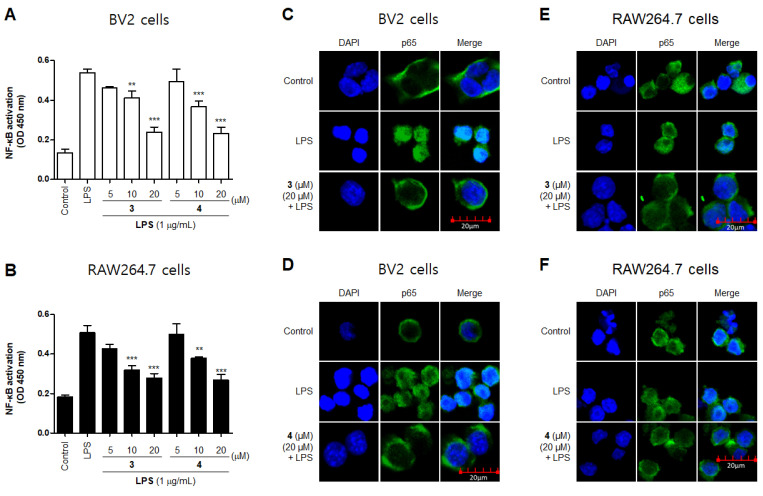
Effects of compounds **3** and **4** on and NF-κB DNA-binding activity (**A**,**B**) and NF-κB (p65) localization (**C**–**F**) in BV2 and RAW264.7 cells. The cells were pretreated with compounds **3** or **4** for 2 h and stimulated with liposaccharide (LPS; 1 μg/mL) for 1 h. Experiments were performed using a commercially available enzyme-linked immunosorbent assay kit, as described in the Materials and Methods section. ** *p* and *** *p* < 0.001 compared with the LPS-treated group.

NF-κB is a transcription factor that regulates iNOS expression [24]. Normal NF-κB remains in its inactive form by forming complexes with regulatory proteins such as IκBα. However, when it is activated by LPS, IκBα is degraded by phosphorylation, and NF-κB (such as p65) is translocated to the nucleus [25], which promotes of the inflammatory mediator gene and induces the expression of inflammatory factors [26]. To further examine the inhibitory effect of compounds **3** and **4** on the production of pro-inflammatory factors in LPS-activated BV2 and RAW264.7 cells, we investigated the expression nuclear translocation of p65 in cells treated with compound **3** and **4**, LPS, or both using an anti-p65 FITC-labeled antibody. DAPI was used for nuclear staining. In the control group, p65 expression was detected in the cytosol. However, in the LPS-induced cells, p65 accumulation was detected in the nucleus, as indicated in the merged images of DAPI and p65 staining. Furthermore, compared with the LPS-induced cells, compounds **3** and **4** markedly reduced the LPS-mediated increase in NF-κB (p65) DNA-binding activity (Figure 6A,B) and nuclear translocation (Figure 6C–F). These findings indicate that compounds **3** and **4** are negative regulators of LPS-stimulated NF-κB nuclear translocation.

### 2.4. Effects of Compounds 3 and 4 on the Nrf2/HO-1 Pathway in BV2 and RAW264.7 Cells

**Figure 7:**
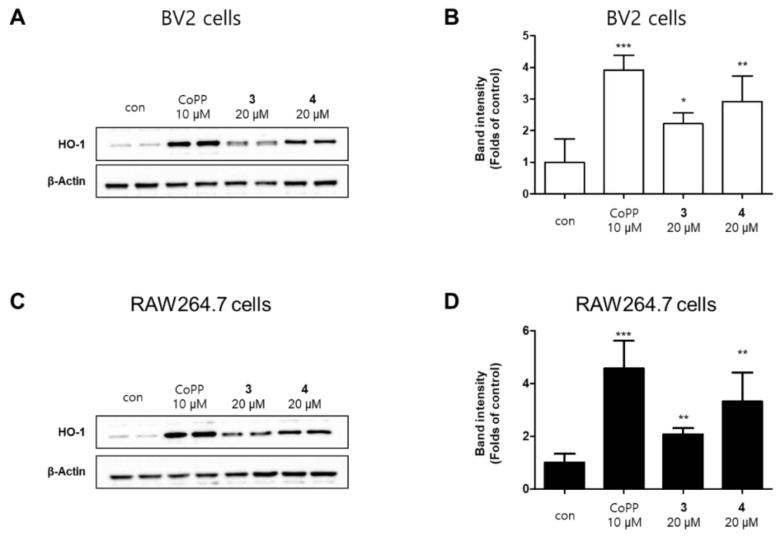
Effects of compounds **3** and **4** on heme oxygenase (HO)-1 expression in BV2 (**A**) and RAW264.7 (**C**) cells. The cells were treated with compounds **3** or **4** or CoPP (10 μM) for 12 h. Representative blots from three independent experiments are shown. Immunoblots were quantified using the ImageJ software. Band intensity was normalized to each total form expression (**B**,**D**). * *p* < 0.05, ** *p* < 0.01, and *** *p* < 0.001 compared with the control group.

**Figure 8:**
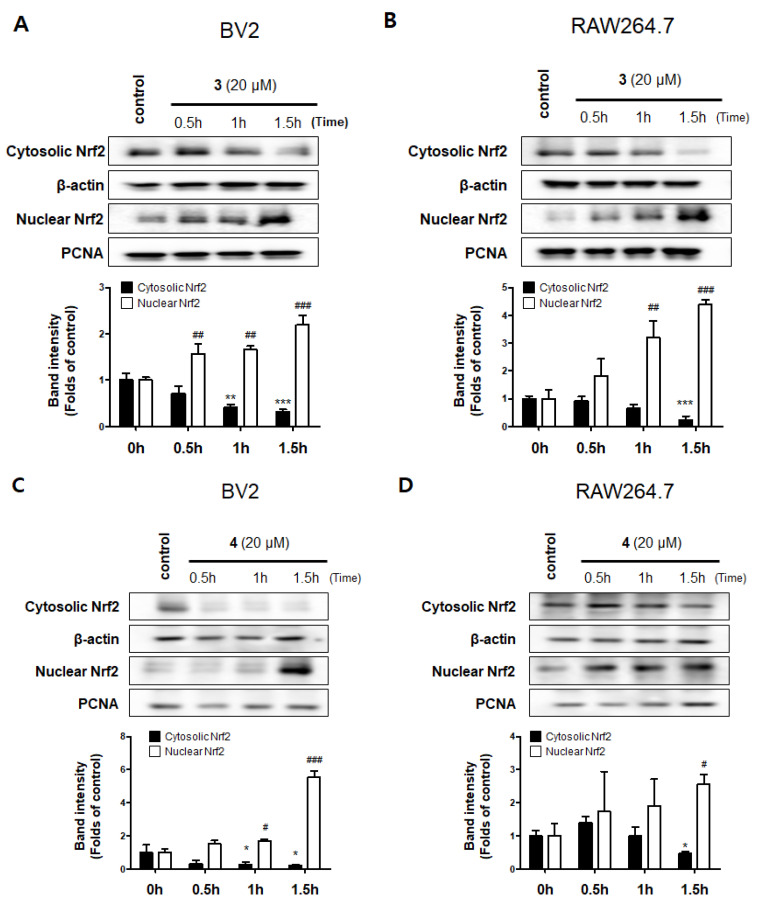
Effects of compounds **3** and **4** on Nrf2 activation in BV2 (**A**,**C**) and RAW264.7 (**B**,**D**) cells. The cells were treated with compounds **3** or **4** for 0.5, 1, and 1.5 h. Representative blots from three independent experiments are shown. Immunoblots were quantified using the ImageJ software. Band intensity was normalized to each β-actin or PCNA. * *p* < 0.05, ** *p* < 0.01, *** *p* < 0.001, # *p* < 0.05, ## *p* < 0.01, and ### *p* < 0.001, compared with the control group.

Hemeoxygenase-1 (HO-1) is a target of nuclear factor E2-related factor 2 (Nrf2), Nrf2/HO-1 pathway is a powerful antioxidant signaling system for promoting free iron in carbon monoxide (CO), billiberdin, and heme [27]. Carbon monoxide, a gaseous metabolite of heme catabolism, exhibits regulatory effects of vasodilation and proinflammatory responses [28]. Besides, HO-1 is also known to play an important role in protecting cells from inflammation and oxidative stress, and to regulate nitrite production in activated macrophages [29]. We performed Western blotting to investigate whether compounds **3** and **4** increase the expression level of HO-1, wherein the well-known HO-1 inducer cobalt protoporphyrin (CoPP) was used as a positive control increasing HO-1 protein expression. [30]. The results revealed that compounds **3** and **4** also upregulated the expression of HO-1 (Figure 7). We further explored whether compounds **3** and **4** regulate the activation of Nrf2. The translocation of Nrf2 to the nucleus was increased in a time-dependent manner, indicating that compounds **3** and **4** significantly upregulated the Nrf2/HO-1 pathway in BV2 and RAW264.7 cells (Figure 8).

**Figure 9:**
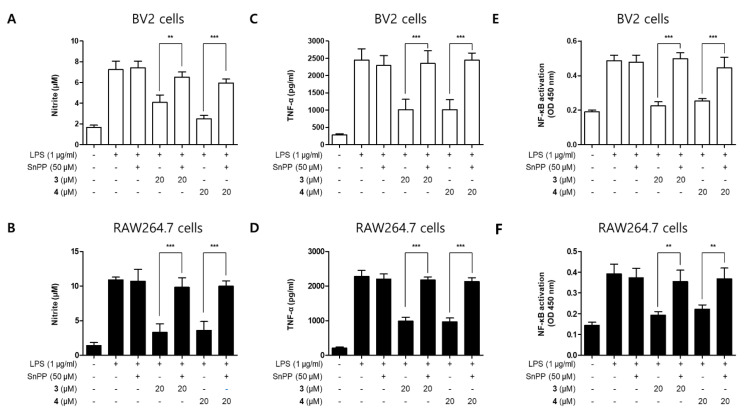
Inhibitory effects of compounds **3** and **4** on nitrite production through the regulation of HO-1 activity in BV2 (**A**,**C**,**E**) and RAW264.7 (**B**,**D**,**F**) cells. The cells were treated with 50 μM of tin protoporphyrin-IX (SnPP) or compounds **3** or **4** and stimulated for 24 h with lipopolysaccharide (LPS; 1 μg/mL). Data are presented as mean ± standard deviation of three independent experiments. ** *p* < 0.01 and *** *p* < 0.001.

To further examine whether the anti-neuroinflammatory and anti-inflammatory effects of compounds **3** and **4** are correlated with HO-1 expression in BV2 and RAW264.7 cells, we performed a set of experiments with tin protoporphyrin-IX (SnPP) which is a selective activity inhibitor of HO-1. Using SnPP, which can downregulate the expression of HO-1, we tried to determine whether HO-1 mediates the inhibitory effect of the above compounds on the inflammatory response induced by LPS [31]. After treating the cells with 20 µM of compounds **3** and **4** for 2 h with or without 50 µM SnPP, they were treated with LPS for 24 h. Although compounds **3** and **4** reduced nitrite production in LPS-induced BV2 and RAW264.7 cells, their anti-inflammatory effects were reversed by SnPP treatment (Figure 9). SnPP alone did not affect nitric oxide (NO) production following LPS stimulation, suggesting that the anti-inflammatory effects of compounds **3** and **4** are regulated by HO-1 expression.

## 3. Discussion

The present study demonstrated that kuwanon T and sanggenon A exert suppressed LPS-induced inflammation in BV2 and RAW264.7 cells. These compounds inhibited the LPS-induced production of NO, PGE_2_, and pro-inflammatory cytokines, including IL-6 and TNF-α, and the expression of iNOS and COX-2 in both BV2 and RAW264.7 cells. These results indicated that kuwanon T and sanggenon A exerted their anti-inflammatory effects by inactivating the NF-κB signaling pathway. In addition, these compounds induced the expression of HO-1 through the activation of Nrf2. In addition, these compounds induced the expression of HO-1 via Nrf2 activation, and this effect was also confirmed to be related to the anti-inflammatory activity.

Nitric oxide synthase (NOS) has three isoforms: neuronal NOS (nNOS; NOS1), iNOS (NOS2), and endothelial eNOS (NOS3) [32]. iNOS is an inducible form that is upregulated in response to various stimuli, including LPS, cytokines, chemokines, and stress, while nNOS and eNOS are constitutive forms that catalyze continuous NO secretion at basal concentrations [33]. Similar to iNOS, COX-2 is also an inducible form upregulated by various inflammatory stimuli, such as cytokines, growth factors, tumor promoters, and bacterial LPS [34]. Its other isoform, COX-1, is constitutively expressed in most tissues under normal physiological conditions [35]. COX-2 exerts an enzymatic effect on the conversion of prostaglandin H2 (PGH2), which converts arachidonic acid to PGE_2_, prostaglandin I2 (PGI2), prostaglandin F2α (PGF_2α_), and thromboxane A2 (TXA_2_) [36]. Under inflammatory conditions, the levels of iNOS and COX-2 increase, resulting in the overproduction of NO and PGE_2_, respectively, and leading to the exacerbation of inflammatory disorders [37]. In the present study, we first evaluated 11 compounds isolated from *M. alba* to determine whether NO production is inhibited in LPS-stimulated in BV2 and RAW264.7 cells. Our results showed that kuwanon T and sanggenon A exerted the strongest inhibitory effects in both BV2 and RAW264.7 cells (Figure 3). In our previous study, moracin M had anti-inflammatory effect on alveolar macrophages, but our results showed a different pattern. However, since each cell has different characteristics, different activities may appear even with the same mechanism [38]. Therefore, as in the results of this paper, it was confirmed that moracin M had no anti-inflammatory activity in BV2 and RAW264.7 cells up to the treatment concentration. Therefore, kuwanon T and sanggenon A were selected for further experiments.

Cytokines have a complex regulatory influence on inflammatory and immune responses [39]. Activation of macrophages and microglial cells enhance the secretion of pro-inflammatory cytokines [40], and this increased secretion leads to further cytokine release [41]. IL-6 is one of the major cytokines and is a soluble mediator responsible for inflammation, immune response, and hematopoiesis [42]. IL-6 signaling is regulated through two different mechanisms, including binding to the membrane-bound IL-6 receptor (mbIL6R) and recognition of the soluble IL-6 receptor (sIL6R) [43]. Both mechanisms are associated with glycoprotein (gp) 130 activation, which results in the activation of downstream signaling molecules, including the Janus kinase (JAK)/signal transducer and activator of transcription (STAT) kinases, phosphoinositide 3-kinase (PI3K), and mitogen-activated protein kinase (MAPK) [44]. IL-6 is used to predict and evaluate inflammation levels in patients with cancer, infection, autoimmune diseases, pancreatic diseases, and cardiovascular diseases.

TNF-α, another major cytokine, also plays an important role in many immune and inflammatory processes, such as immune cell proliferation, apoptosis, necrosis, and survival. The biological effects of TNF-α are regulated by binding to two different receptors, tumor necrosis factor receptor (TNFR)1 and TNFR2. TNFR1 is the main mediator of TNF-α activity in most cells, because TNFR2 has less binding affinity for TNF-α, resulting in TNF-α dissociating more easily from TNFR2 than from TNFR1. Abnormalities in TNF-α signaling and overproduction of TNF-α lead to the development of many diseases, including rheumatoid arthritis, psoriasis, Crohn’s disease, atherosclerosis, sepsis, diabetes, and obesity [45,46]. Therefore, it is important to inhibit the production of pro-inflammatory cytokines to suppress and prevent inflammatory reactions and the development of inflammatory diseases. In the present study, kuwanon T and sanggenon A inhibited the LPS-induced production of IL-6 and TNF-α in both BV2 and RAW264.7 cells.

NF-κB is the most ubiquitous transcription factor [47], and NF-κB signaling plays an important role in the expression of genes related to inflammatory responses, including those encoding iNOS, COX-2, and pro-inflammatory cytokines [48]. In the inactivated state, NF-κB subunits exist in the cytoplasm bound to its inhibitor protein, inhibitor kappa B (IκB)-α. Several stimuli, including LPS and cytokines, induce the phosphorylation and degradation of IκB-α, allowing the liberation and translocation of NF-κB subunits into the nucleus. The translocated subunits bind to κB sites of target genes in DNA, resulting in the induction of transcription of genes encoding pro-inflammatory mediators [49]. Therefore, inactivation of the NF-κB pathway may be a therapeutic target for inflammatory diseases. In the present study, pretreatment with kuwanon T and sanggenon A inhibited the LPS-induced activation of NF-κB signaling by inhibiting the DNA-binding activity and nuclear translocation of NF-κB subunits (Figure 6). These results suggest that the anti-inflammatory effects of kuwanon T and sanggenon A are exerted via regulation of the NF-κB signaling pathway.

Under normal conditions, Nrf2 binds to Kelch-like ECH-associated protein 1 (Keap1) in the cytoplasm. However, it dissociates from Keap1, translocates into the nucleus, and binds to antioxidant response element (ARE) sites on the DNA, leading to the expression of various antioxidant genes, including those encoding HO-1, NAD(P)H:quinone oxidoreductase 1 (NQO1), peroxiredoxin (Prx), and thioredoxin (Trx) [50]. In particular, HO-1 is known to be associated with anti-inflammatory effects, and its activity is represented by the levels of CO, one of the three by-products generated from heme by the enzymatic activity of HO-1 [51]. In the present study, we found that kuwanon T and sanggenon A induced HO-1 expression by activating Nrf2 (Figure 7 and Figure 8). In addition, we verified the correlation between the anti-inflammatory effect of kuwanon T and sanggenon A and HO-1 expression using SnPP, a selective inhibitor of HO-1 activity. The inhibitory effects of kuwanon T and sanggenon A on NO and TNF-α production and NF-κB activation were partially reversed by co-treatment with SnPP (Figure 9). These results suggest that the anti-inflammatory effects of kuwanon T and sanggenon A are regulated by HO-1 expression.

## 4. Materials and Methods

### 4.1. Materials

Roswell Park Memorial Institute 1640 (RPMI 1640) and fetal bovine serum were purchased from Gibco BRL Co. (Grand Island, NY, USA). All chemicals were obtained from Sigma-Aldrich Chemical Co. (St. Louis, MO, USA). The primary antibodies anti-iNOS, anti-β-actin, anti-p65 and anti-HO-1 were purchased from Santa Cruz Biotechnology (Santa Cruz, CA, USA); and anti-rabbit and anti-mouse secondary antibodies from Millipore (Billerica, MA, USA). Enzyme-linked immunosorbent assay (ELISA) kits for PGE_2_, IL-6, and TNF-α were purchased from R&D Systems Inc. (Minneapolis, MN, USA). The isolation and structural determination of the 11 compounds from *Morus alba* have been described elsewhere [19,20,21].

### 4.2. Cell Culture and Viability Assays

BV2 and RAW264.7 cells were seeded at a density of 5 × 10^5^ cells/mL in RPMI 1640 supplemented with 1% antibiotic (penicillin–streptomycin) and 10% heat-inactivated FBS. The cells were cultured at 37 °C in a humidified 5% CO_2_ with 95% air atmosphere, according to a previously described method [52].

### 4.3. Measurement of NO Production

The production of nitrite, a stable end-product of NO oxidation, was measured as an indicator of NO production in cells. Briefly, the concentration of nitrite in the conditioned media was determined using a method based on the Griess reaction [53]. The details of the assay have been described previously [54].

### 4.4. PGE_2_ Assay

The concentration of PGE_2_ in each sample was measured using a commercially available ELISA kit, according to a previously described method [55].

### 4.5. Measurement of IL-6 and TNF-α Levels

The culture medium was collected to determine the levels of IL-6 and TNF-α using a commercially available kit (BioLegend, San Diego, CA, USA). The assay was performed according to the manufacturer’s instructions. Briefly, BV2 and RAW264.7 cells were seeded in 24-well culture plates at a density of 20 × 10^5^ cells/well. After incubation, the supernatant was collected and used to measure the concentrations of IL-6 and TNF-α with ELISA kits.

### 4.6. Western Blot Analysis

The pelleted BV2 and RAW264.7 cells were washed with PBS and lysed in RIPA buffer. Equal amounts of proteins were quantified using protein assay dye reagent concentrate obtained from Bio-Rad Laboratories (#5000006; Hercules, CA, USA), mixed in the sample loading buffer, and separated using SDS-PAGE. The separated proteins were then transferred onto a nitrocellulose membrane. Non-specific binding to the membrane was blocked by incubation in a solution of skim milk. The membrane was incubated with primary antibodies (all of which were used at a 1:1000) at 4 °C overnight and then reacted with a horseradish peroxidase-conjugated secondary antibody (all of which were used at a 1:5000) (Millipore) [31].

### 4.7. NF-κB Localization and Immunofluorescence Analysis

To study the localization of NF-κB, BV2 and RAW264.7 cells were cultured on Lab-Tek II chamber slides and treated with different concentrations of compounds for 2 h before LPS stimulation (0.5 μg/mL) for 1 h. The cells were then fixed in formalin and permeabilized with cold acetone and probed with anti-p65 antibodies (1:200), followed by incubation with a fluorescein isothiocyanate-labeled secondary antibody (1:1000) (Alexa Fluor 488, Invitrogen). To visualize nuclei, cells were treated with 1 μg/mL of 4′,6-diamidino-2-phenylindole (DAPI) for 30 min, washed with PBS for 5 min, and treated with 50 μL VectaShield (Vector Laboratories, Burlingame, CA, USA). Stained cells were visualized and images were acquired using a Zeiss fluorescence microscope (Provis AX70; Olympus Optical Co., Tokyo, Japan). [31].

### 4.8. Statistical Analysis

Data are presented as mean ± standard deviation of three independent experiments. One-way analysis of variance, followed by Tukey’s multiple comparison test, was used to compare differences among the three groups. Statistical analyses were performed using the GraphPad Prism software (version 5.01, GraphPad Software Inc., San Diego, CA, USA).

## 5. Conclusions

Kuwanon T and sanggenon A exerted anti-inflammatory effects in LPS-induced BV2 and RAW264.7 cells by inhibiting the production of NO, PGE_2_, IL-6, and TNF-α and the expression of iNOS and COX-2. Our results demonstrated that these inhibitory effects were mediated via inactivation of the NF-κB pathway by treatment with kuwanon T and sanggenon A. In addition, these compounds induced the expression of HO-1 by activating the translocation of Nrf2 into the nucleus. Our findings demonstrated that the HO-1 expression induced by kuwanon T and sanggenon A was associated with the suppressive effects against LPS-induced inflammation. Taken together, our results provide evidence that kuwanon T and sanggenon A isolated from *M. alba* could be candidates for the development of therapeutic and preventive agents for neuroinflammatory diseases such as Alzheimer’s disease and Parkinson’s disease.

## Data Availability

The data presented in this study are available within the article. Other data that support the findings of this study are available upon request from the corresponding author.

## Supplementary Materials

The following are available online. Figure S1: Inhibitory effects of compounds **3** and **4** on nitrite production in BV2 (A,C) and RAW264.7 (B,D) cells.

## Sample Availability

Not available.
