# Supplementary material for: Kuwanon T and Sanggenon A Isolated from Morus alba Exert Anti-Inflammatory Effects by Regulating NF-κB and HO-1/Nrf2 Signaling Pathways in BV2 and RAW264.7 Cells
Source: Molecules. 2021 Dec 16;26(24):7642. doi: 10.3390/molecules26247642 (PMC8708433; doi:10.3390/molecules26247642)
Supplement: Supplementary file 1 [file molecules-26-07642-s001.zip › molecules-1478064-supplementary.pdf]

Article

Kuwanon T and sanggenon A isolated from *Morus alba* exert anti-inflammatory effects by regulating NF-κB and HO-1/Nrf2 signaling pathways in BV2 and RAW264.7 cells

Wonmin Ko [1,#], Zhiming Liu [1,#], Kwan-Woo Kim [2,#], Linsha Dong [1], Hwan Lee [1], Na Young Kim [3], Dong-Sung Lee [1,*] and Eun-Rhan Woo [1,*]

1 College of Pharmacy, Chosun University, Dong-gu, Gwangju, 61452, Republic of Korea; rabis815@naver.com (W.K.); lzmqust@126.com (Z.L.); donglinsha011@163.com (L.D.); ghksdldi123@hanmail.net (H.L.); dslee2771@chosun.ac.kr (D.S.L.); wooer@chosun.ac.kr (E.R.W.)
2 Department of Herbal Crop Research, National Institute of Horticultural and Herbal Science, RDA, Eumseong 27709, Republic of Korea; swamp1@naver.com (K.W.K.)
3 Pathology Research Division, National Institute of Fisheries Science, Busan 46083, Republic of Korea; pharm001@korea.kr (N.Y.K)
# Wonmin Ko, Zhiming Liu, and Kwan-Woo Kim contributed equally to this work.
* Correspondence: dslee2771@chosun.ac.kr; wooer@chosun.ac.kr; Tel.: +82-62-230-6386, Fax: +82-62-222-5414

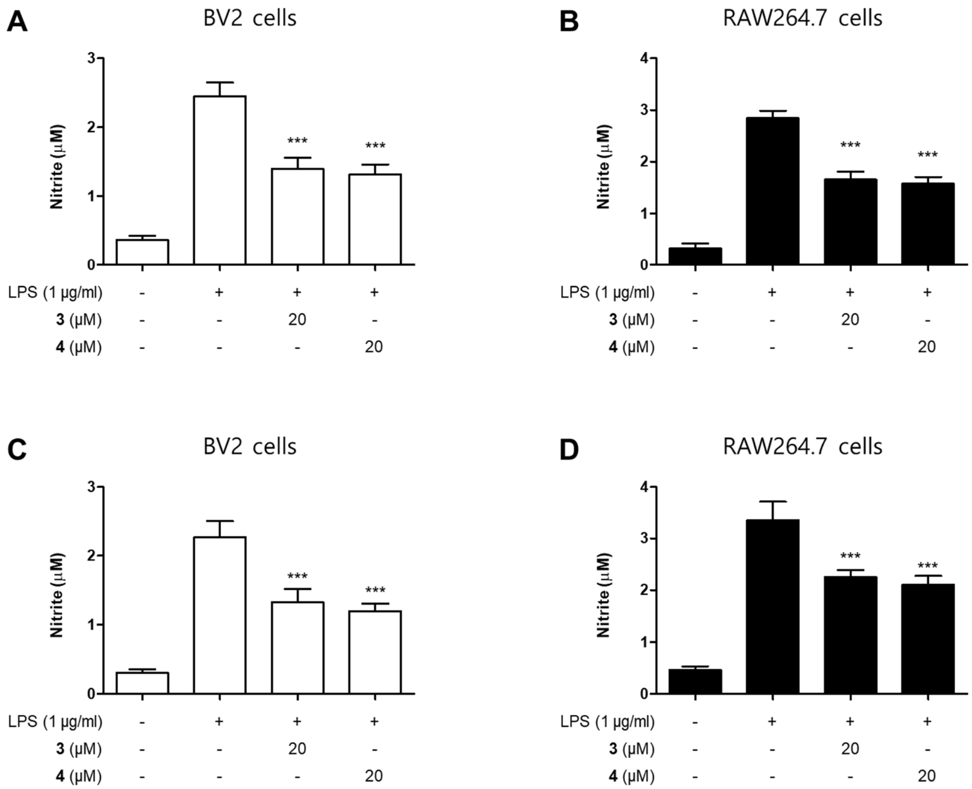

Supplementary Figure S1. Inhibitory effects of compounds **3** and **4** on nitrite production in BV2 (A,C) and RAW264.7 (B,D) cells. (A and B): The cells were pretreated for 2 h with concentrations of compounds and stimulated for 24 h with lipopolysaccharide (LPS; 1 μg/mL). (C and D): The cells were pretreated for 2 h with LPS (1 μg/mL) and stimulated for 24 h with concentrations of compounds. Error bars represent mean ± standard deviation of three independent experiments. ***$p < 0.001$ compared with the LPS-treated group.
